# ITC study on the interaction of some bile salts with tragacanth, Arabic, and guar gums with potential cholesterol-lowering ability

**DOI:** 10.3389/fnut.2023.1258282

**Published:** 2023-10-24

**Authors:** Michele Massa, Carlotta Compari, Emilia Fisicaro

**Affiliations:** ^1^Department of Maternal Infantile and Urological Sciences, Sapienza University of Rome, Rome, Italy; ^2^Department Food and Drug, University of Parma, Parma, Italy

**Keywords:** cholesterol-lowering ability, soluble dietary fiber, Arabic gum, tragacanth gum, guar gum, bile salt, soluble dietary fiber-bile salt interaction, functional food

## Abstract

**Introduction:**

The urge of designing new safe and natural functional foods to control blood lipids and dispensable without the need of physician supervision, has increased especially after the coming into effect of the recent EU Commission regulation 2022/860, that regulates the consumption of “red yeast rice,” made by fermentation of rice with *Monascus purpureus*, and perceived as a natural functional food, due to a health risk for frail consumers. The results of the present work are a part of the systematic study we are carrying out of the binding ability of some soluble dietary fibers (SDF) from different natural sources toward selected bile salts (BS).

**Methods:**

Measurements were carried out by isothermal titration calorimetry (ITC) with the idea to shed light on the mechanism, if any, by which they show cholesterol-lowering activity.

**Results and discussion:**

Epidemiological studies are sometimes conflicting and offer only hypothesis about the mechanism of action, the most accredited being the reduction of reabsorption of BS in the gut. Previous measurements done on negatively charged pectin and alginate, showed specific binding interaction with monomer NaDC for pectin and no interaction at all for alginate. Chitosan, positively charged and soluble only at low pH, in 100 mM acetate buffer at pH = 3 shows strong exothermic interactions with NaTC and NaTDC. Here we considered two plant exudates (Arabic gum and tragacanth gum) and guar gum, extracted from guar beans, and their interaction with the same bile salts. ITC measurements do not evidence specific interactions between gums and the studied BS, so that their cholesterol lowering ability, if any, is due to a different mechanism very probably bound to the viscosity increase. Moreover, the addition of NaC, the most abundant BS in the bile, at very low concentration (under the cmc) causes a structural change of the solution. The obtained results seem to corroborate the hypothesis that the cholesterol lowering activity is related to the increase in viscosity of guar solution favored by NaC, the major component of the bile.

## 1. Introduction

Hypercholesterolemia, characterized by elevated blood cholesterol levels, is a silent disease that significantly impacts the lives of individuals. It is the major risk factor for cardiovascular diseases, the leading cause of mortality in developed countries (1, 2). Pharmacological treatments based on fibrates and statins are commonly used to reduce high blood cholesterol in adults, but the associated side effects often lead to suboptimal adherence to the therapy (3, 4). Therefore, there is a growing interest in identifying natural and biocompatible dietary supplements that can effectively control low-density lipoprotein (LDL) cholesterol levels, for both prevention and treatment of hypercholesterolemia, particularly in children, through dietary habit modifications.

Recent authorization by the European Medicines Agency (EMA) of bempedoic acid, a novel cholesterol-lowering drug with a different mechanism of action than statins, has further highlighted the importance of exploring alternative approaches (EMA/65186/2020). Moreover, the introduction of EU Commission regulation 2022/860 in June 2022, which regulates the use of red yeast rice (RYR), emphasizes the need for natural and safe dietary supplements for cholesterol control (5). RYR, traditionally used in China both as food colorant and to foster digestion and blood circulation, is obtained through the fermentation of rice with *Monascus purpureus* yeast, resulting in the production of monacolins, including monacolin K (also known as lovastatin) (6). Due to the natural process by which it is obtained, RYR was perceived as a natural nutraceutical product, suitable also for frail people, such as statin-intolerant people and children (7, 8), and used to prevent lifestyle-related diseases by improving dietary habits (1, 5–9). However, RYR carries risks similar to statin drugs due to the presence of monacolins, making it potentially harmful to certain vulnerable groups (10) and, in addition, the content in monacolins is variable, depending on the fermentation process and on the raw material (10), making difficult the observance of the correct dose. In addition, an incorrect choice of the raw material or an incorrect fermentation process could cause the presence of citrinin, a mycotoxin produced also by *Monascus purpureus* and known for nephrotoxic and hepatotoxic properties (11). Therefore, exploring alternative natural compounds with cholesterol-lowering potential, such as soluble dietary fibers (SDF), has become crucial.

SDFs, abundantly found in fruits and vegetables, have been recognized for their ability to enhance the cholesterol-lowering effects of diets (12–15). As complex carbohydrates resistant to human enzyme hydrolysis, SDFs are not digested or absorbed by the human body. Instead, they undergo fermentation by gut bacteria, producing short-chain fatty acids that aid in reducing blood cholesterol via various mechanisms (2). Epidemiological studies have consistently shown the health benefits of consuming soluble fibers, supporting their potential role in disease prevention (2, 16–20). Although the cholesterol-lowering effect of SDF has been hypothesized for over 40 years, the exact chemical mechanisms underlying their activity are still under debate. One hypothesis involves the interaction between specific SDF and bile salts, potentially through specific binding, which prevents their reabsorption in the gastrointestinal tract. Consequently, the liver is stimulated to synthesize new bile salts from cholesterol, thus maintaining cholesterol homeostasis (12, 16–19, 21, 22). Bile salts (BS) play a crucial role in intestinal lipid absorption and cholesterol homeostasis as about 50% of cholesterol degradation occurs via its catabolism to form bile acid (23). Bile is produced by the liver, stored in the gall bladder, and released into the small bowel in response to a meal to favor the digestion and absorption of dietary lipids. It is normally recovered in the terminal ileum and recycled, up to several times within a meal (24).

With the goal of creating a pool of SDF with optimal cholesterol-lowering ability for the development of functional foods, we undertook a systematic study using isothermal titration calorimetry (ITC) to investigate the binding ability of SDF from different natural sources with various bile salts (12). Functional foods are defined as whole, fortified, enriched, or enhanced foods that have a potentially beneficial effect on health when consumed as part of a varied diet (25, 26). Previous results, discussed in detail in (12), focused on the interaction of pectin, alginate, and chitosan with sodium cholate (NaC), sodium deoxycholate (NaDC), sodium taurocholate (NaTC), and sodium taurodeoxycholate (NaTDC). These investigations revealed that the binding ability varies depending on the specific SDF and bile salt pair, suggesting a specific and selective behavior rather than a generic SDF-bile salt interaction. In this article, we present the results obtained using the same technique and bile salts, but with Arabic gum, tragacanth gum, and guar gum as the SDF. Arabic gum and tragacanth gum are natural exudate gums released from plants under external stress or injury and find applications in many sectors from food to non-food industries (27). Guar gum, on the other hand, originates from guar beans, containing galactomannan gum, which forms a gel in water (28).

## 2. Materials and methods

### 2.1. Materials

Sodium cholate hydrate (NaC), sodium taurocholate hydrate (NaTC), sodium deoxycholate (NaDC), and sodium taurodeoxycholare (NaTDC) from Sigma-Aldrich were used as received. Fifty millimeter solutions of these BS were prepared using boiled doubly distilled water.

Arabic, tragacanth, and guar gums were supplied by ACEF, Fiorenzuola d'Arda (PC, Italy), and were used as received. 1% w/v SDF solutions were prepared by weight using boiled doubly distilled water and were stirred overnight at a constant rate.

### 2.2. Isothermal titration calorimetry

ITC measurements were carried out using a MicroCal PEAQ-ITC (Malvern) set to a temperature of 30°C at which the micelle formation enthalpy is small for all the investigated BS (12). The BS solution was injected (first injection of 0.4 μL, followed by 18 injections of 2 μL each or 25 injections of 1.5 μL each) into a 200 μL reaction cell filled with an SDF solution 1 g L^−1^ using a 40 μL automatized syringe with a 120 s interval between two successive injections. Continuous stirring at 150 rpm was maintained throughout the experiments. Data analysis was performed using MicroCal PEAQ-ITC Analysis Software (version 1.41, Malvern Panalytical, Malvern, UK).

## 3. Results and discussion

**Figure 1:**
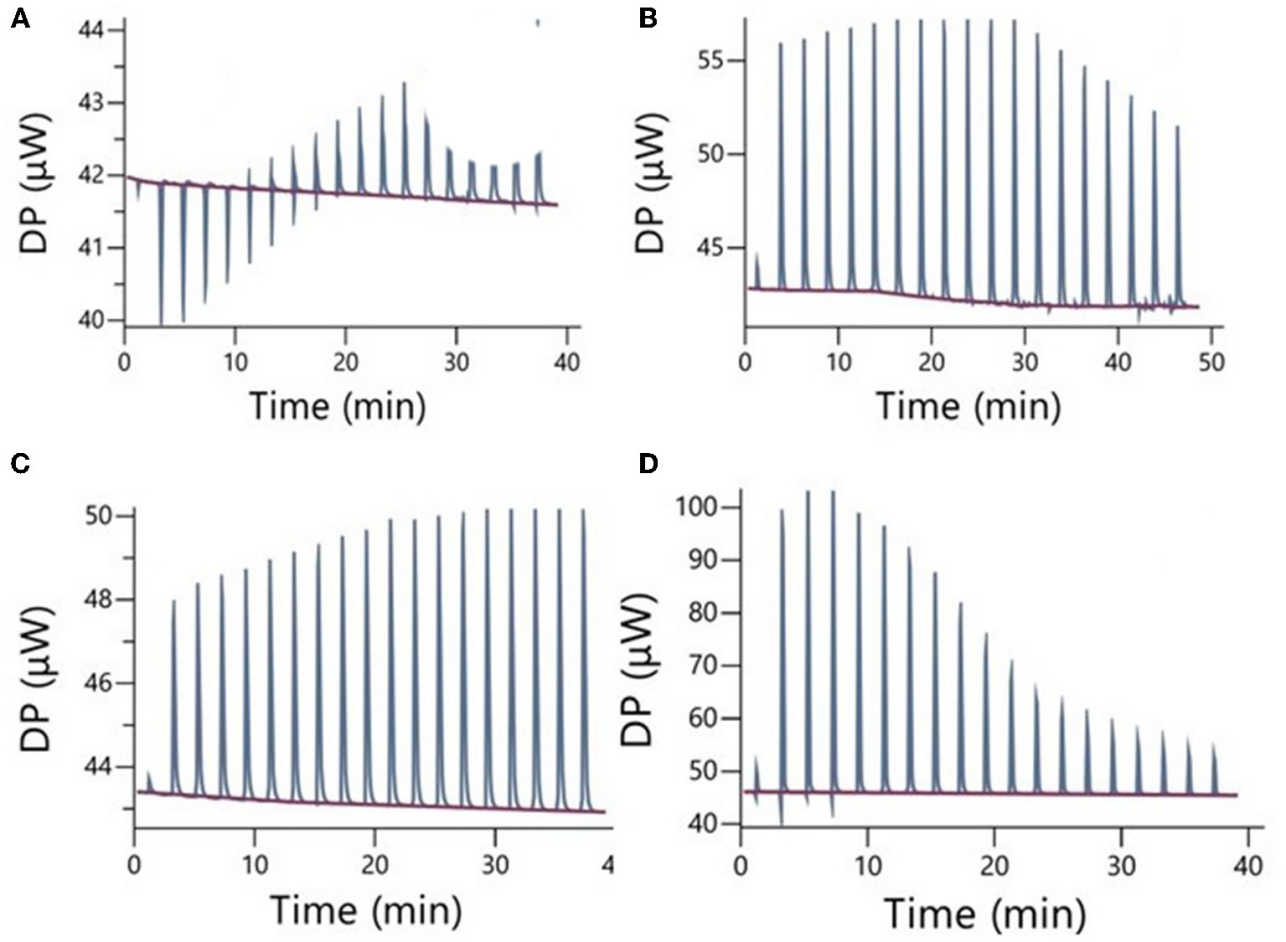
Raw dilution heats of the bile salts in the same conditions as interaction experiments: **(A)** NaC 50 mM; **(B)** NaDC 50 mM; **(C)** NaTC 50 mM; **(D)** NaTDC 50 mM. These dilution heats must be subtracted from the raw interaction heats, reported in the next figures, to obtain the true interaction heat.

**Figure 2:**
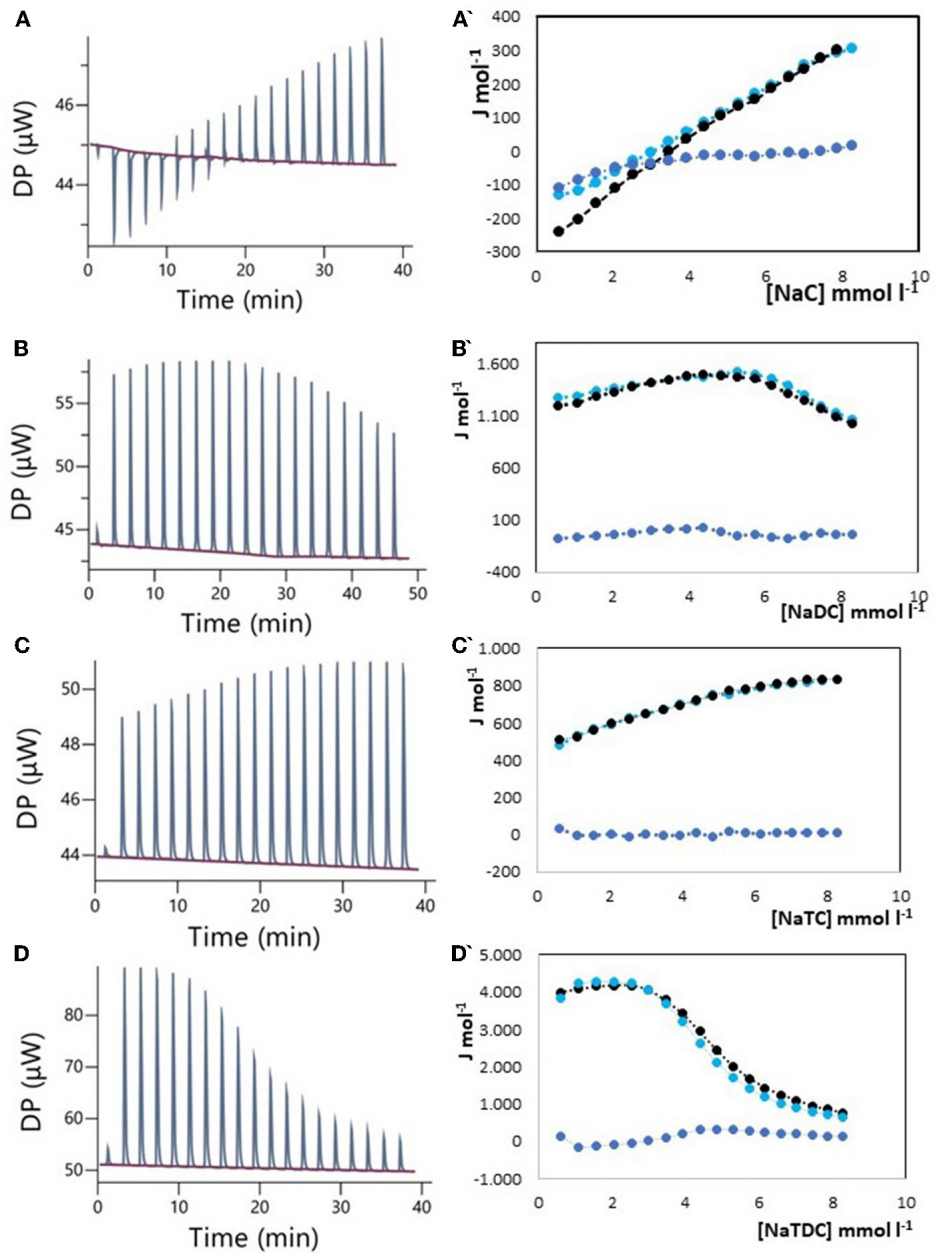
Raw heat rate (μJ s^−1^) vs. time profiles obtained from injection of titrant (first injection of 0.4 μL, followed by 18 injections of 2 μL each) into a 200 μL reaction cell filled with an Arabic gum solution 1 g L^−1^ (blue, in red the baseline), and (specified by a single quote mark) the dependence of the enthalpy change vs. bile salt concentration in the reaction cell for: **(A)** NaC 50 mM; **(B)** NaDC 50 mM; **(C)** NaTC 50 mM; **(D)** NaTDC 50 mM. Black dots represent the interaction enthalpy between pectin and bile salt, the light blue dots represent the dilution enthalpies of bile salt and the blue dots represent their difference.

**Figure 3:**
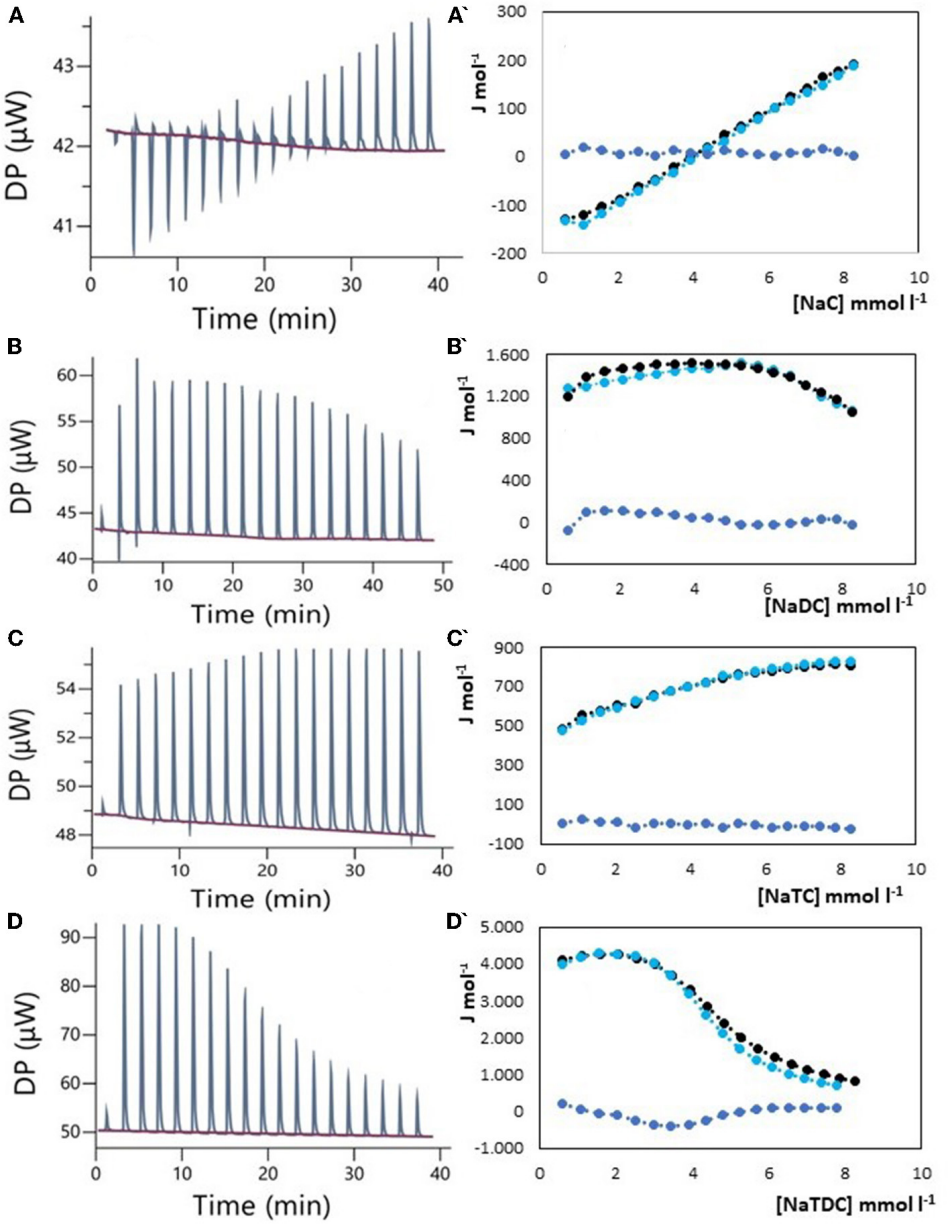
Raw heat rate (μJ s^−1^) vs. time profiles obtained from injection of titrant (first injection of 0.4 μL, followed by 18 injections of 2 μL each) into a 200 μL reaction cell filled with a tragacanth gum solution 1 g L^−1^ (blue, in red the baseline), and (specified by a single quote mark) the dependence of the enthalpy change vs. bile salt concentration in the reaction cell for: **(A)** NaC 50 mM; **(B)** NaDC 50 mM; **(C)** NaTC 50 mM; **(D)** NaTDC 50 mM. Black dots represent the interaction enthalpy between pectin and bile salt, the light blue dots represent the dilution enthalpies of bile salt, and the blue dots represent their difference.

**Figure 4:**
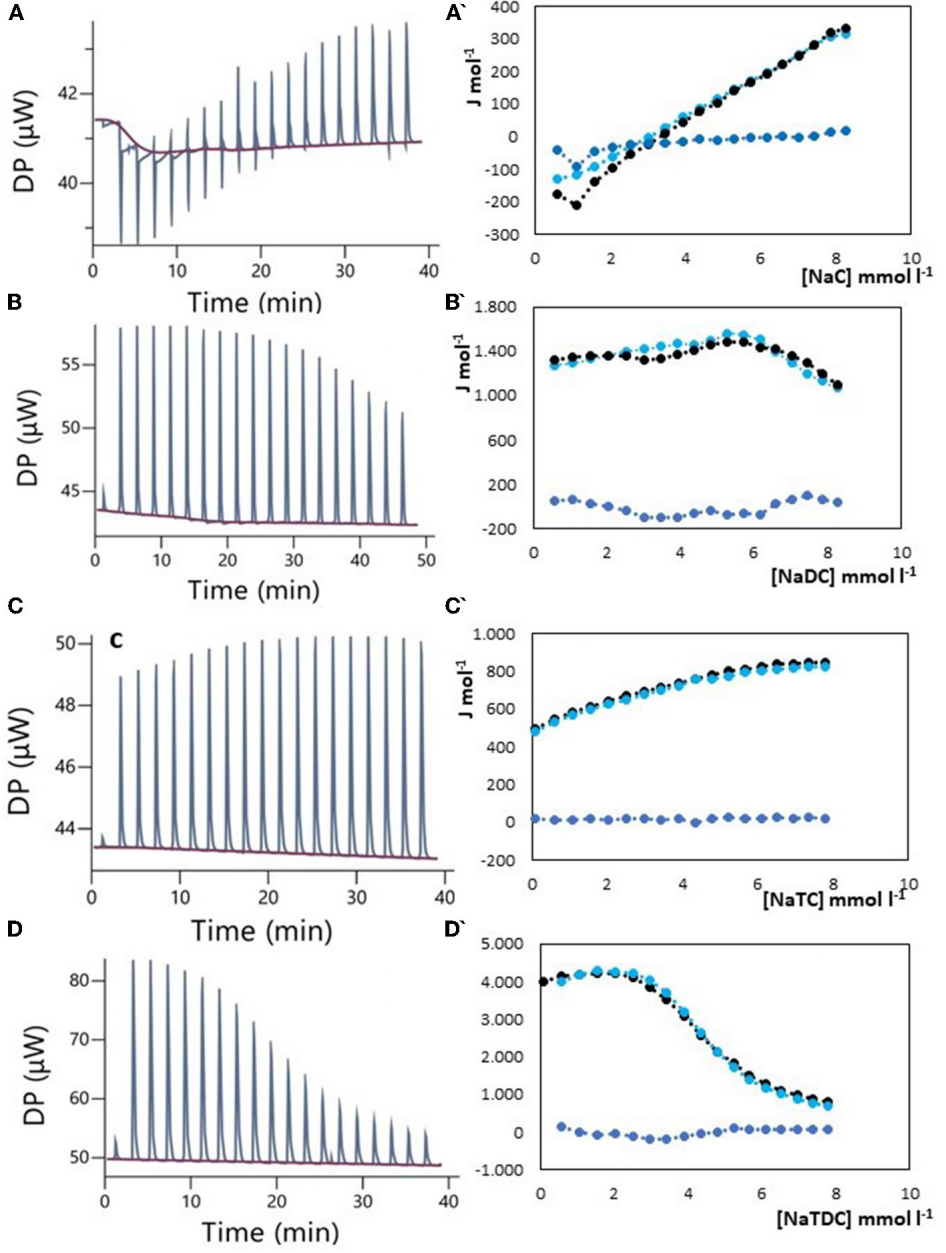
Raw heat rate (μJ s^−1^) vs. time profiles obtained from injection of titrant (first injection of 0.4 μL, followed by 18 injections of 2 μL each) into a 200 μL reaction cell filled with a guar gum solution 1 g L^−1^ (blue, in red the baseline), and (specified by a single quote mark) the dependence of the enthalpy change vs. bile salt concentration in the reaction cell for: **(A)** NaC 50 mM; **(B)** NaDC 50 mM; **(C)** NaTC 50 mM; **(D)** NaTDC 50 mM. Black dots represent the interaction enthalpy between pectin and bile salt, the light blue dots the dilution enthalpies of bile salt and the blue dots their difference.

The results of ITC measurements are shown in Figures 1–4. In Figure 1, the raw dilution heats of the bile salts under study obtained in the same conditions as interaction experiments are shown. These dilution heats, originating from the disruption of BS micelles, must be subtracted from the raw interaction heats with SDF solutions to obtain the net interaction heat. In panels A–D of Figures 2–4 we present the row data from ITC titrations, whereas panels A'–D' display the heats obtained by integration of the peaks as a function of titrant concentration. Specifically, the blue peaks in panels A–D derive from the titration of SDF 1 gL^−1^ by 50 mM BS solutions. In panels A'–D' the raw integrated interaction heats are indicated by black dots, the integrated dilution heats by light blue dots, and their difference, the true interaction heat, by blue dots. BS are natural surfactants having in the same molecule a hydrophobic and a hydrophilic surface, at a difference of common surfactants constituted by a polar head and by one, or more, hydrophobic tails. The chemical structures of the BS used in this work are shown in Figure 3 of (12). Due to their structure, they form micelles, with a low aggregation number (29, 30), able to emulsify fats during digestion. The process of micelle formation or micelle disruption is associated with an exchange of heat with the surroundings. The 50 mM BS solutions we used as titrants are at a concentration greater than their critical micelle concentration (cmc) (12). As a result, when these solutions are introduced into the calorimetric cell where they are diluted, the micelles break down exchanging heat with the surroundings. The knowledge of the energetics of the micelle formation process is, therefore, the starting point for the study of the interaction of BS with SDF. We have already discussed this point in depth in a previous paper (12) to which we refer for details. Here we recall that ITC dilution experiments provide information about the critical micelle concentration, cmc, and the thermodynamic parameters of micellization by the same experiment (12–31). Generally, the dihydroxy bile salts (NaDC and NaTDC) have lower cmc values compared to the trihydroxy salts (NaC and NaTC) due to their lower hydrophobicity resulting from the lack of a hydroxyl group. As is typical for all hydrophobic processes, the micellization enthalpy depends on temperature. We used a temperature of 30°C to minimize this heat contribution and reach a greater sensitivity in our measurements (12). BS dilution experiments were made by ITC in the same conditions as the interaction with SDF, using water in the calorimetric cell and BS solution in the syringe. The heat released in dilution experiments must be subtracted from the raw heat obtained when the BS solution (in the syringe) is added to the SDF solution in the cell, to obtain the correct net interaction heat.

### 3.1. Bile salts interaction with Arabic gum

Arabic gum, derived from Acacia Senegal and Acacia Seyal trees, is the oldest of all natural gums: it was used by the ancient Egyptians back to the third millennium B.C. (32). In contemporary times, it is widely used as a stabilizer, a thickening agent, and an emulsifier mainly in the food, cosmetic, and pharmaceutical industry (27, 33, 34). Its chemical composition is quite complex and dependent on source, tree age, climatic conditions, and soil environment. Arabic gum is a branched-chain polysaccharide, with a backbone composed of 1,3-linked b-D-galactopyranosyl units, either neutral or slightly acidic, due to the presence of a polysaccharidic acid (arabic acid) and its salts with calcium, magnesium, and potassium (33). There are only a few epidemiological studies on the cholesterol-lowering ability of acacia gum yielding conflicting results. Recently, the effect of Arabic gum on metabolic syndrome has been evaluated by a single-blind, randomized, placebo-controlled trial (34). It appears that, although Arabic gum has some beneficial effects for people at risk of metabolic syndrome, there are no changes in blood lipid profile. On the contrary, in the same year, another study showed that Arabic gum can significantly decrease body weight, blood glucose, total cholesterol, and low-density lipoprotein in mice fed with a high-fat diet (35). Farman et al. (36) evaluated the effect of gum Arabic administration in chronic renal failure patients. They found a significant decrease in blood cholesterol and triglycerides (34). The evaluation of the interaction between Arabic gum and BS by isothermal titration calorimetry (ITC) could help to clarify this point. ITC is, in fact, a powerful tool to provide a number of important thermodynamic parameters about the equilibrium under study, without the need for sample pretreatment, and it has wide applications in food science (12, 37). Due to the high sensitivity, ITC measurements can provide information about specific interactions in complex systems such as those we are now considering. In Figure 2, the ITC results obtained by titrating a 1 g L^−1^ solution of Arabic gum with 50 mM solutions of (A) NaC, (B) NaDC, (C) NaTC, and (D) NaTDC are shown. Specifically, Figures 2A–D shows (i) the heat rates (μJ s^−1^) *vs*. time profiles obtained by injecting a 50 mM solution of the different BS (first injection of 0.4 μL, followed by 18 injections of 2 μL each or 25 injections of 1.5 μL each) into a 200 μL reaction cell filled with an Arabic gum solution 1 g L^−1^, and (ii) (specified by a single quote mark) the enthalpy change of interaction between Arabic gum and BS (black dots), dilution enthalpy of BS (light blue dots) and their difference (blue dots) vs. BS concentration in the reaction cell, obtained by integration of the peaks. The dilution heat of the SDF in the calorimetric cell resulted under the detection limits of the instruments and was disregarded. The net interaction heat between Arabic gum and BS, represented by the blue dots in Figure 2, does not differ significantly from zero, in the limits of the experimental error, indicating the absence of site-specific interactions. It must be outlined that the concentration reached in the calorimetric cell at the end of titration is lower than cmc (it is greater than the cmc only for NaTDC) since a 50 mM concentration of BS was used as the titrant. In this way, we can exclude specific interactions with BS in monomeric form [the most important specific interaction for pectin and chitosan (12)] for all the studied BS and as micelles only for NaTDC. While this finding rules out BS binding as a mechanism for cholesterol-lowering activity, it does not exclude other mechanisms, such as an increase in solution viscosity, which may play a role. However, it should be noted that Arabic gum is a highly branched polymer, forming particles nearly spherical, resulting in a limited solution viscosity. A recent review with the scope to resolve enduring misconceptions about insoluble and soluble fibers (24), claims, in contrast with our findings, that only high viscous SDF (e.g., gel-forming fibers) can lower elevated serum cholesterol concentrations, a health benefit, therefore, viscosity dependent. Both these aspects, the absence of specific interaction and the low viscosity, could suggest the limited effectiveness of Arabic gum as a cholesterol-lowering agent, although its emulsifying properties remain noteworthy.

### 3.2. Bile salts interaction with tragacanth gum

Tragacanth gum, an ancient polysaccharide known since the time of Theophrastus, shares some similarities with Arabic gum. It is a natural exudate obtained from the stem of the bush-like plant of the Astragalus species and finds applications in the food and pharmaceutical industries (38–42). Unlike Arabic gum, tragacanth gum forms highly viscous solutions and is considered the most viscous of natural plant gums (42). While there are no claims regarding the cholesterol-lowering activity of tragacanth gum in the literature, it is outlined its high viscosity and potential in green chemistry (38), biomedical engineering (41), and as a prebiotic in functional foods (39). The high viscosity of the aqueous solutions could explain the experimental difficulties we encountered in obtaining an acceptable ITC thermogram. Tragacanth gum has in its structure many carboxylic groups that make the viscosity vary with ionic strength and pH. Maximum viscosity is typically achieved at pH 5–6, which is the natural pH of the gum when dissolved in water, and viscosity decreases as the pH decreases due to reduced carboxylic group dissociation (42, 43). Results of our ITC studies, reported in Figure 3 and performed in the same way as for Arabic gum, confirm the absence of specific interactions between the gum and the BS examined.

### 3.3. Bile salts interaction with guar gum

Guar gum, derived from Cyamopsis tetragonoloba beans, is a cold-water soluble polysaccharide composed of mannose and galactose units. It is widely used as a food additive for emulsification, thickening, and processed food solidification purposes (28, 44, 45). Clinical trials investigating the effects of guar gum supplementation on blood lipids have yielded mixed results, particularly in studies conducted in the late 20th century. A 2007 review of the epidemiological studies of the inclusion of guar gum in the diet (28) of both humans and animals, defined the guar gum as a “miracle therapy” for hypercholesterolemia, hyperglycemia, and obesity. A very recent meta-regression and dose–response meta-analysis of randomized placebo-controlled trials on the effect of guar gum in adults indicated that guar gum supplementation may have favorable effects on total cholesterol (TC) and low-density lipoproteins (LDL) without significant alterations in total triglycerides (TAG) and high-density lipoproteins (LDL) (44). The mechanisms by which guar gum lowers TC and LDL are still hypothetical and lack experimental evidence (28, 46). It makes sense, therefore, to evaluate the ability of guar gum to bind BS, without disregarding the high viscosity reached by its solutions. The results, shown in Figure 4, indicate that NaDC, NaTC, and NaTDC did not exhibit specific interactions with guar gum. However, the behavior of the system with NaC requires a more detailed explanation. We encountered experimental difficulties at the beginning of the titration, with occasional anomalous spikes not attributable to specific interactions, as in previous observations in pectin-NaDC or chitosan-NaTC/NaTDC systems (12). These spikes were likely caused by non-reproducible disturbances during the measurement (see Supplementary Figure 1), and we tentatively ascribe to a local change in viscosity following the addition of NaC. In the absence of these spikes, there was a small difference between the interaction heat and the dilution heat of NaC, indicating the absence of site-specific interactions but suggesting something occurring in the solution. One possible explanation is, in fact, an increase in solution viscosity due to the addition of NaC, within a concentration range where the BS is still in its monomeric form (before the cmc). The titrations reported in Figure 4A, A' are made by using as titrant NaC 50 mM, so that at the end of titration its concentration is still under the cmc. Figure 4A clearly shows the instability of the baseline (in red) during the initial injections of NaC, not found for the other BS studied. We performed also some titration using NaC 200 mM for exploring the micellar region (see Supplementary Figure 2) and verify if there was a change in NaC cmc, indicating that a given quantity of NaC, bound to the guar gum, is subtracted from the micellization equilibrium. The value of the cmc resulted always the same indicating that NaC activity remains unchanged, excluding a binding interaction with guar gum. The obtained results seem to corroborate the hypothesis that the cholesterol-lowering activity is related to the increase in viscosity of the guar solution favored by NaC, the major component of the bile.

## 4. Conclusion

The intake of dietary fibers has been associated with numerous health benefits, as supported by epidemiological studies and recent meta-analyses. The need for new, safe, and natural functional foods to control blood lipids, has become increasingly important, especially after the coming into effect of the recent EU Commission regulation 2022/860, which mandates the consumption of “red yeast rice” only under the supervision of a physician, due to health risks for some consumers. The results of the present study are a part of our ongoing systematic study on the binding ability of SDF from different natural sources toward selected bile salts by isothermal titration calorimetry (ITC), which aims to shed light on the mechanism underlying their cholesterol-lowering activity. Insights from this study can contribute to the design of new functional foods incorporating a combination of fibers possessing enhanced cholesterol-lowering properties. This paves the way for the development of safe and effective dietary strategies to reduce blood cholesterol levels and lower the risk of cardiovascular diseases (CVD), even for vulnerable populations.

Previous measurements carried out on two negatively charged SDFs, such as pectin and alginate, showed specific binding interaction with monomer NaDC for pectin and no interaction at all for alginate. Chitosan, positively charged and soluble only at low pH, in 100 mM acetate buffer at pH = 3 shows strong exothermic interactions with the bile salts soluble at this pH (NaTC and NaTDC) with precipitate formation This study investigated the interactions between BS and two plant exudates (Arabic gum and tragacanth gum) and guar gum, extracted from guar beans. The results do not evidence specific interaction between gums and the studied BS. Hence, if these gums exhibit cholesterol-lowering effects, an alternate mechanism might be at play, possibly linked to the viscosity increase of the solution. Specifically, the study revealed that the addition of NaC, the most abundant bile salt, at very low concentrations (below the critical micelle concentration) induced a structural change in the guar gum solution.

Future research should explore the mechanisms underlying the cholesterol-lowering effects of SDFs in greater detail. This may involve investigating the impact of SDFs on cholesterol absorption, bile acid synthesis, hepatic cholesterol metabolism, and the gut microbiome.

The chemical information obtained from this study, combined with epidemiological evidence, can contribute to the development of functional foods that marry high cholesterol-lowering abilities with an appealing taste profile. These foods hold promises as both preventive and therapeutic supplements, improving patient compliance and overall health outcomes.

## Data availability statement

The raw data supporting the conclusions of this article will be made available by the authors, without undue reservation.

## Author contributions

MM: Conceptualization, Data curation, Methodology, Supervision, Writing—original draft, Writing—review and editing. CC: Resources, Writing—review and editing. EF: Conceptualization, Data curation, Methodology, Supervision, Writing—original draft, Writing—review and editing.
