# Supplementary material for: ITC study on the interaction of some bile salts with tragacanth, Arabic, and guar gums with potential cholesterol-lowering ability
Source: Front Nutr. 2023 Oct 24;10:1258282. doi: 10.3389/fnut.2023.1258282 (PMC10628474; doi:10.3389/fnut.2023.1258282)
Supplement: Supplementary file 1 [file Data_Sheet_1.PDF]

Supplementary Material

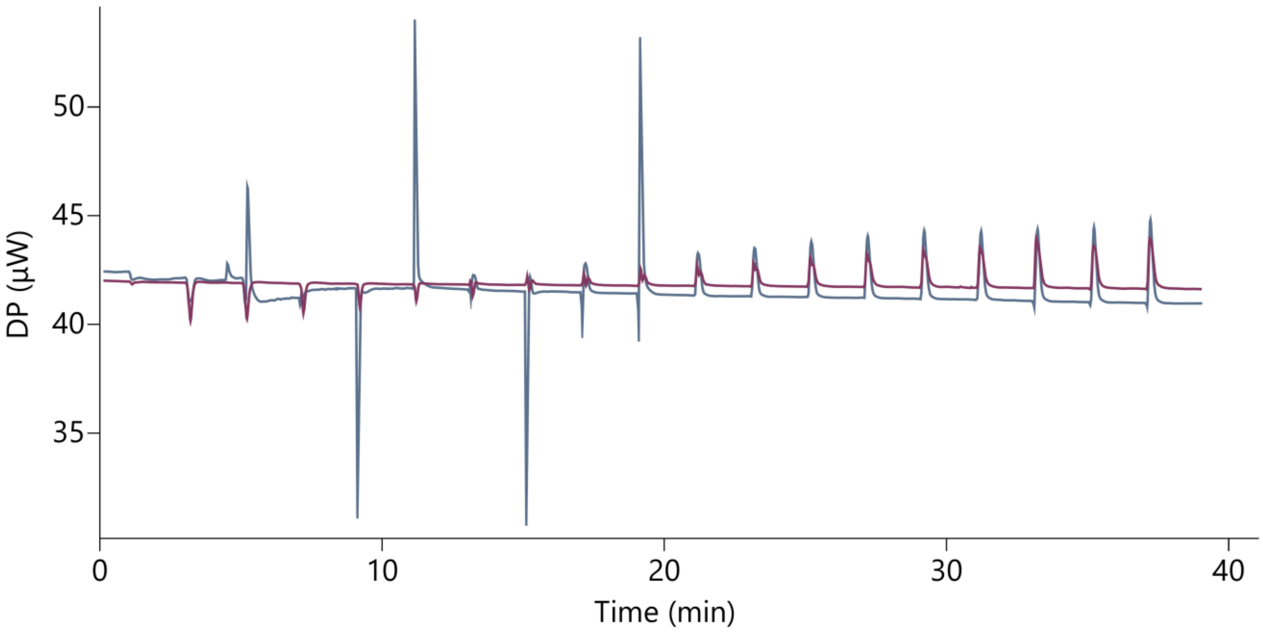

Supplementary Figure 1. Heat rate (μJ s^{-1}) *vs*. time profiles obtained from injection of titrant (first injection of 0.4 μL, followed by 18 injections of 2 μL each) into a 200 μL reaction cell filled with a guar gum solution 1 g L^{-1} (black) compared with the dilution of NaC 50 mM (red) in the same conditions. The occasional anomalous spikes not attributable to specific interactions, are shown.

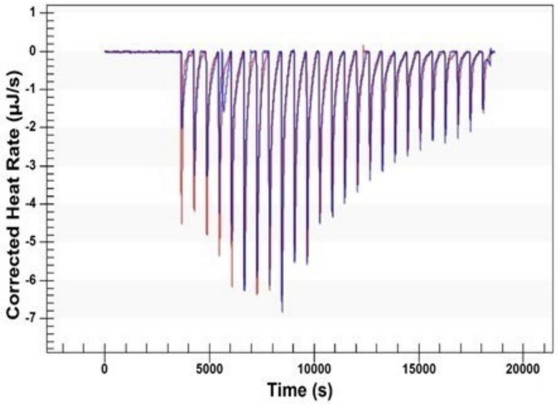

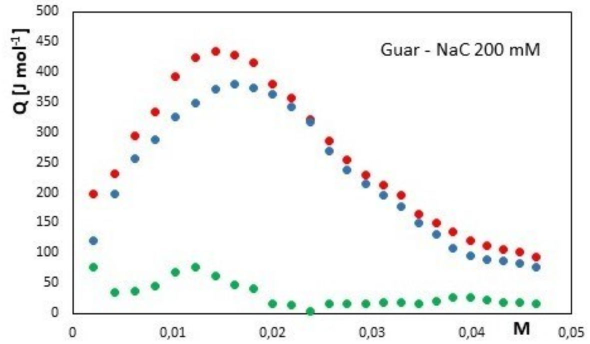

Supplementary Figure 2. Left: Heat rate (µJ s^{-1}) *vs*. time profiles obtained from 25 injection each of 10 µL into a 960 µL reaction cell filled with guar gum solution 1 g L^{-1} (red) compared with the dilution of NaC 200 mM (blue) in the same conditions, and right: the dependence of the enthalpy change vs. NaC concentration in the reaction cell. Red dots represent the interaction enthalpy between guar gum and NaC, the blue dots the dilution enthalpies of bile salt and the green dots their difference. ITC measurements were carried out on a CSC model 5300 N-ITCIII isothermal titration calorimeter (Calorimetry Sciences Corporations, USA) at 30°C.
